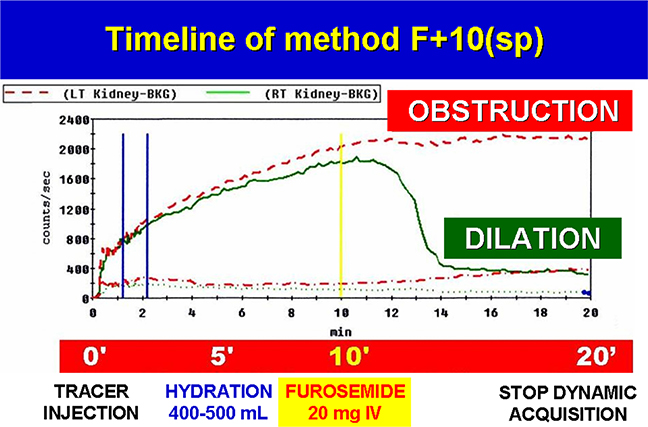# Diuresis renography in equivocal urinary tract obstruction. A historical perspective

**DOI:** 10.1016/j.biopha.2019.108981

**Published:** 2019-05-25

**Authors:** Girolamo Tartaglione, Danyelle M. Townsend, Pier Francesco Bassi, Roberto C. Delgado Bolton, Francesco Giammarile, Domenico Rubello

**Affiliations:** a Service of Nuclear Medicine, Cristo Re Hospital, Rome, Italy; b Department of Drug Discovery & Pharmaceutical Sciences, Medical University of South Carolina, USA; c Department of Urology, Catholic University of Sacred Heart, Rome, Italy; d Department of Nuclear Medicine, Universidad de La Rioja, Spain; e International Atomic Energy Agency (IAEA), Nuclear Medicine and Diagnostic Imaging Section, Vienna, Austria; f Department of Nuclear Medicine, Radiology, Neuroradiology, and Clinical Pathology, Santa Maria della Misericordia Hospital, Rovigo, Italy

**Keywords:** Hydronephrosis, Dynamic renal radiotracers, Diuresis renography, Furosemide, Gravity

## Abstract

Obstructive nephropathy may be suspected for the incidental detection of dilated renal collecting system at ultrasonography, CT or MRI. A dilated renal collecting dilation (calyco-pelvis or ureteres) might be related 1) to an anatomical variant of the excretory tract without obstruction and, therefore, without consequence on renal function, or 2) to an obstruction/stenosis of the urinary tract that may cause a damage of kidney function. In the present review we annotated the various methods proposed for Diuresis Renography (DR) used with the purpose to make early diagnosis of obstructive nephropathy. First, the F+20 method (i.e. furosemide 40 mg injected IV 20 min after radiotracer injection) in seated position (sp) (F+20_(sp)_) was reported to distinguish between an anatomical dilation from an anatomical obstruction of the urinary tract. It was also suggested to perform DR with the patient in supine or prone position in order to minimize possible furosemide-induced hypotension and patient’s movements during exam. Other DR methods were proposed administering furosemide EV to the patient in supine position at different times: F-15 (furosemide injected IV 15 min prior to radiotracer), F0 (furosemide injected contemporary to radiotracer), F+20 (furosemide injected 20 min after the radiotracer), F-20 and Well Tempered (other than F+20 this modality requires saline infusion for all duration of the test plus bladder catheterization). Unfortunately, in all the above described DR methods with patientin supine position, despite the furosemide administration, a sensitive slowing down of urinary outflow could be related to the supine position itself of the patient during the examination. Lastly, there are reports of a new DR method based on furosemide IV injection 10 min after radiotracer with the patient in seated position, F+10_(sp)_. This method allows a better timing between hydration (400 mL of water) at 5 min, and the injection of relatively low dose of furosemide (20 mg), thus avoiding side effects as diuretic-induced hypotension and favouring bladder filling, therefore ameliorating patient compliance and reducing equivocal responses.

## 1. Introduction

Dilation of the urinary tract is commonly discovered during abdominal ultrasonography (US), CT or MRI typically in the absence of clinical signs and symptoms. In some cases, the dilated renal collecting system depends on an anatomical obstruction/stenosis associated with a progressive atrophy of the renal cortical tissue and a lowering of renal function: in these patients a dis-obstructive intervention may be required to preserve and possibly to ameliorate renal function [1]. In other cases, dilation of the renal pelvis and calyces is not associated with obstruction/stenosis of renal tract but is simply related to an anatomical variant characterized by a greater compliance of calico-pelvis system in the absence of obstruction/ stenosis [2]. The need for a more accurate diagnostic tool capable of accurately distinguishing an obstructive from a non-ostructive calyco-pelvis dilation and to evaluate kidney function in order to timely address the patient to a conservative or surgical approach, has rapidly increased over the last years, especially in relation to the large availability of surgical approaches [3–7].

Generally, diuresis renography (DR) is preferred as diagnostic tool, in comparison to gadolinium MRI, in differential diagnosis of dilation of renal collecting system because it allows: a) to measure the relative renal function, b) to evaluate the diuretic-stimulated washout of the tracer, avoiding the use of gadolinium), c) moreover, providing a very low radiation exposure with possibility to study also infants [8].

## 2. Historical analysis of Diuresis Renography (DR)

### 2.1. The F+20 method in seated position (F+20_(sp)_)

In 1978, O’Reilly and colleagues [9] introduced DR F+20_(sp)_ as a protocol of choice for the evaluation of the outflow from the urinary tract. Initially, the renography was performed with the patient in a sitting position and in normal state of hydration using a dual probe renogram positioned on the backside close to kidneys, following IV infusion of I^131^Hippuran. In the event renography showed an obstructive pattern with little or no excretion, acquisition was haulted for 4–5 minutes while the patient consumed 500 mL of water or juice and allowed to void the bladder. Appoximately 20 min after the tracer injection (F+20), the patient received 40 mg furosemide (IV) and a second dynamic acquisition was obtained for 20 min. If renal radioactivity remained unchanged after furosemide treatment, anatomical obstruction/stenosis was positively confirmed. On the other hand, if renal radioactivity significantly decreased after the injection of the diuretic this was consistent with a perviety of the urinary tract [9]. In the same studies, O’Reilly evaluated an alternate strategy to perform the F+20 test whereby furosemide IV was injected during data acquisition, without an interruption of 4 min to void the bladder [9]. With the RD F+20_(sp)_ protocol an absent or a good response is observed in ^~^ 85% of cases, while in the other 15% the response to diuretic was suboptimal. Subsequently, these investigators showed a modified version of the RD F+20 method resulted in an equivocal rate in15% of patients [10]. Originally the F+20 procedure was performed with the patient in a seated position, however in 1987 a survey conducted in UK revealed a wide variation in techniques used for routine renography: the majority of renography practitioners (56%) used supine position, 32% seated position, and 12% other positions (prone or semirecumbent) [11].

These data were related to the fact that many clinicians preferred the supine position to reduce movements of the patient and the risk of diuresis-induced hypotension. On the other hand, the supine position might cause a physiological radiotracer stasis in the dilated pelvis even in the absence of obstruction. As a consequence, the supine position may increase the number of false positive or equivocal studies.

In an effort to improve fidelity, the protocol was further modified by English et al. [12] who implemented the DR F-15 method. This approach leveraged findings that the maximum effect of furosemide was observed 10–15 minutes after injection. In general, 40 mg furosemide (IV) increases the urinary flow from 1 to 3 mL/min, to an average value of 24 mL/min. Hydration was necessary for optimal results and as such patients were given 400–500 mL of water 30 min prior. Then, 15 min before tracer injection, 40 mg furosemide was administered IV while the patient was in the supine position, in order to reduce the risk of diuretic-related hypotension. After voiding, I^123^ Hippuran was injected and the renogram began with the patient in seated position, using a gamma camera-computer system. The quantitative evaluation of this modified DR was based on an index of excretion: the T1/2 which represent the time from the peak of the renogram until the activity in the kidney falls to 50% of its maximum value. Normally the T1/2 ranges 5 to 10 min. A T1/2 value greater than 10 min indicates obstruction. The F-15 method has been considered more specific than the F+20 [13]. However, the F-15 method did not provide information about the basal renal function, so it might be used as a secondary follow-up exam.

In 1992, an important recommendation was delivered from joint meetings of The Society for Fetal Urology, The Pediatric Nuclear Medicine Council, and The Society of Nuclear Medicine, in order to ameliorate DR. The modified protocol, Well-Tempered F+20 method, was particularly useful in paediatrics and incorporated a strategy based on: a) patient preparation, b) radiotracer used, c) patient position during examination, d) data acquisition, e) diuretic administration and dosage, f) time of injection, g) regions of interest (ROI) in which monitoring the diuretic effect, h) data analysis [14]. The Well-Tempered F+20 procedure was performed in the supine position in well-hydrated patients in order to improve renal function and avoid risk of diuretic-induced hypotension. An IV line was inserted for the administration of saline solution at a rate of 15 mL/kg during a 30-min interval and beginning 15 min prior to inject the radiotracer: usually the ^99m^Tc-MAG3. The saline infusion continued throughout the study at a liquid volume rate of 200 cc/kg/24 h. If the drainage phase of renogram was slowed the furosemide was injected IV at a dose of 1 mg/kg at 20th min (F+20) and examination continued for another 20 min. Another important aspect of Well-Tempered F-20 renogram was catheterization of the bladder following saline infusion to assure 1) adequate drainage throughout the study, 2) to reduce absorbed radiation dose to the bladder and gonads, and 3) to avoid patient movements due to impending urination. This protocol allowed continuous evaluation while eliminating the false positive interpretation of test related to an empty bladder empty. If the scintigraphic images suggest that the pelvis or ureter were incompletely drained at the termination of the diuretic renogram phase, the pediatric patient was placed in the prone position. The interpretation of these results were based on visual patterns analysis of the ^99m^Tc-MAG3 time-activity diuresis phase renogram curves. Four outcomes were described: *Normal, No activity, Indeterminate, and Obstruction*. Among the index for calculating drainage, the 20 min/peak ratio and clearance half-time of the radionuclide from the renal pelvis during the diuresis phase were considered. In conclusion the protocol underlined the exigency of a strict collaboration of urological and nuclear medicine practitioners, that might improve the management of pediatric patients with an obstructive nephropathy, reducing the number of equivocal or false positive study.

In 1999, The Society and Nuclear Medicine guidelines v. 2.0 suggested the use of prone or supine position during evaluation, underlining that caution should be observed with postural changes due to possible diuresis-induced hypotension [15]. In the European community, many authors reported the Well Tempered F+20 RD was not easy to apply to ambulatory young patients. Consequently, Wong and colleagues proposed the RD F0 protocol based on the simultaneous administration of ^99m^TcMAG3 and furosemide 1 mg/Kg in order to simplify the exam [16]. The test was performed in the supine position and offered many advantages, including time savings and less invasive relative to other protocols, resulting in better patient compliance and reduced number of tests disrupted because of voiding. The disadvantages of early-furosemide injection were a loss of information about baseline renal function and the influence on split renal function (right Vs. left renal function).

The need for an additional scan after gravity-assisted drainage (GAD) in DR was emphasized by Rossleigh et al. [17]. At the end of the 20-min diuretic phase, a 5 min post- GAD image acquisition after voiding and in erect position was recommended. The percentage of residual activity was calculated by comparison of post void scan with the last 5 min of the supine diuretic phase [18]. In this way, patients were upright to void and the gravity outflow urinary drainage from a dilated but not from an obstructed collecting system. When the test was performed in supine position, it was strongly suggested that a later post voiding static image should obtained after upright posture irrespective of the timing of the furosemide injection [19]. The Society of Nuclear Medicine guidelines v. 3.0 (2008) confirmed the need for a later acquisition before and after the patient was maintained in upright position for 10–15 minutes [20]. The post-voiding images should be erect if possible and should always be obtained at the conclusion of the study to reduce equivocal results. These findings were widely supported. In 2011, the International Scientific Committee on Radionuclide Nephrourology (ISCORN) confirmed that the renal drainage might normalize after upright posture and voiding, and very often the findings of late Post Micturition (PM) image might contradict the information provided by T_1/2_ calculated in supine position [21].

The European Association of Nuclear Medicine (EANM) guidelines for children recommended to perform DR(sp) in 2001. Several protocols have been reported giving furosemide at different time: F-15, F0, F+2, F+20 (Table 1) but accordingly to the EANM guidelines there was no evidence that one method was superior to the others [22]. Each method had several advantages: the DR F+20 method provided information about baseline renal function, but was characterized by 15% equivocal results at least; the DR F-15 method was considered to be the most specific but lacked data on renal function in baseline conditions; the RD F0 method was more practical than others, resulting in fewer interrupted studies to void [23,24]. Early furosemide tests (F-15 and F0) were considered to have deficiency as they lack information about baseline renal function conditions. The simultaneous administration of furosemide with the tracer induced an early acceleration of renal transit. This was considered when calculating renal function separately, for example by favouring the integral method [25]. Some studies that used the Patlak-Rutland slope method recommended injecting furosemide 2 min after tracer injection (F+2) [26]. In an inter-observer reproducibility study, it was shown that a wide range of interpretations about drainage were conducted by nuclear physicians [27]. The reasons of these divergences were the absence of a clear limit between partial and good or almost good drainage, and the fact of including or neglecting the effect of micturition and change of patient’s position.

Quantitative parameters, estimated on the first two minutes and on the late PM images, might help to improve thestandardisation in interpreting the RD renogram and the quality of drainage data [28]. In calculating the relative (Right Vs. Left kidney) and the absolute renal uptake of ^99m^Tc-MAG3, inter-operator variability in the assignment of the renal region of interest (ROI) drawn around kidney and background are critical factor. A semi-automated method of assigning the renal ROI has been implemented [29]. A gamma camera method with no blood sampling was proposed to calculate differential renal function (DRF) based on initial renal uptake, and required an appropriate background correction using automated background ROIs surrounding each kidney area [30]. The dynamic sequence of renography allows estimation of Split Renal Uptake % (Right Vs. Left kidney in %) and the Differential Renal Function in ml/min (DRF) [31,32]; this was calculated within the first 2 min of the renogram. A DRF of 45–55% between Right and Left Kidney was considered to be within the normal range. DRF measures relative function hydronephrotic kidney and is dependent upon the entity of tracer extraction and might reflect changes in function of the opposite kidney. Drainage was classically described using the T1/2 defined as the time for half the accumulated tracer to leave pelvis. Prolonged T_1/2_ may have been influenced by many factors including: poor renal function that might affect the descending part of renogram, a reduced peristalsis of pelvis, bladder fullness and obviously the effect of gravity.

An empty bladder and gravity assume increased importance when there is delayed drainage from a supine patient or if there are abnormal peristalsis. Therefore, drainage of pelvis should be evaluated by simple methods considering gravity and empty bladder, which are less influenced by renal function such as Pelvic Excretion Efficiency (PEE), Output Efficiency (OE) and Normalized Residual Activity (NORA) [33]. These parameters allowed quantification of the renal drainage at any moment throughout renographic acquisition. OE can then determined and expressed as a percentage of the zero-output curve (the curve that would have been obtained if no activity had left the kidney) [34]. NORA is defined as the renal activity at a given moment (end of renogram, end of furosemide acquisition, and a later image after micturition) divided by the renal activity between 1 and 2 min. The 90th percentile values of NORA are, in the normal group, 0.70 at 20 min, 0.23 at the end of the furosemide test, and 0.10 after micturition at 50–60 min after tracer injection irrespective of the timing of diuretic. Two variables that might influence the results of NORA are the choice of background correction and an error in the estimation of the 1–2 min renal activity. The standardization of both parameters is mandatory to be able to compare the results obtained at different centers. In all cases a very similar quality of drainage was reached when considering the Post Micturition (PM) or Post Voiding (PV) image, that remains mandatory to obtain irrespective of the timing of the furosemide injection.

A later PM (or PV) image with NORA evaluation was strongly recommended by EANM guidelines. NORA was obtained after micturition between 50 and 60 min after tracer injection independently of timing of the diuretic injection [35]. This included the effect of gravity because the patients were encouraged to void and walk for few minutes after the end of the furosemide acquisition. A Guidance Document for Structured Reporting of Diuresis Renography published by ISCORN Committee confirms the importance to state in the report all modalities in which was executed the test as hydration (oral or intravenous), radiotracer, dose and timing of furosemide, method of clearance measurement (camera based, single plasma sample, multiple plasma samples), the presence of a bladder catheter, urinary diversion or nephrostomy tube, and of course the patient position during acquisition. The document provides a basic structure and rationale for a standard DR report that should document the results in a clear manner; contain the essential elements required to interpret the study; encourage clinical research by facilitating better comparison of results between institutions [36]. A recent document published by EANM (2016) encourages the use of seated position (sp) in obstructive renal pathology. The acquisition in the erect position can be preferable because of the hydrostatic pressure, because more realistic results will be achieved, document confirms [37].

Recently a new DR F+10(sp) method has been proposed [38] to facilitate the diagnosis of obstructive nephropathy in adult patient. The patients were studied in erect (seated) position with patient’s back close to gamma camera and in normal state of physiological hydration. A dose of ^99m^Tc-MAG3 was injected at time 0′ and dynamic acquisition started in a seated position for 20 min. The subjects were administered 400–500 mL of water orally 5 min after the tracer injection. A lower dose of furosemide (20 mg) is given IV at 10 min after tracer injection during renogram acquisition (Table 2). Later PM images in both seated and supine position were obtained to complete the examination after dynamic acquisition and 60′ after tracer injection, as illustrated in. Usually, bladder catheter and saline solution infusion are not requested. These tests has the advantage of providing information about baseline renal function, and the split renal function (Right Vs. Left renal in %); moreover, was not influenced by early furosemide or hyperhydration and can distinguish between normal pelvis (Tmax < 7 min) and dilated (Tmax > 7 min). The procedure was based on a better timing between hydration and the IV administration of a reduced dose of furosemide. The F+10(sp) protocol avoids the side effects like bladder filling and diuretic induced hypotension. The evaluation of test was based on the measure of *Split Renal Function* (normal value = 0.50 +/−10), *Time to Peak* (normal value < 7 min.), *Diuretic T*_*1/2*_ (normal value < 8 min.), *Ratio 20min/peak* (normal value < 0.25), and comparison between post voiding images at 20 and 60 min in seated and supine position [39]. This approach gave a higher significance to the drainage index similar to a *20 min/Peak Ratio*, facilitating better comparison of the results. In addition, comparing early uptake images 1–2 min in a seated position with later supine scan can facilitate and diagnose renal ptosis, making clear the influence of the nephroptosis on the renal function and drainage phase.

Comparing DR F+10(sp) with DR F-15 methods in supine position, in the same group of patients, a lower incidence of equivocal results were observed. DR F+10(sp) was a useful alternative to antegrade/ retrograde pyelogram in patients with a percutaneous nephrostomy or in bladder cancer patients with urinary diversion and reconstruction, allowing a correct evaluation of urinary drainage in a physiological way. DR F+10(sp) reduced the equivocal cases and thanks to the semiquantitative drainage index improves the objectivity of test. In March 2017, the DR F+10(sp) method was approved as national guideline by Italian Association of Nuclear Medicine (AIMN v. 3/2017) [40]. Also current British Nuclear medicine Society (BNMS) guidelines recommends to study patients in an erect position, seated in a suitable imaging chair, because it offers a sufficient support to prevent the patient from moving during the study [41]. In a recent paper for Continuing Education of Society of Nuclear Medicine and Molecular Imaging (SNMMI), Taylor recommended the method F+10(sp), underlining the importance of gravity-assisted drainage regardless of the timing of furosemide [42].

Under the auspices of the International Atomic Energy Agency (IAEA) a “Software Package for the Analysis of Scintigraphic Renal Dynamic Studies” was recently developed. The implementation of IAEA Software Package enables the normal limits of quantitative ^99m^Tc-MAG3 parameters to be established and would improve the standardization of renography and facilitate the comparison of reports between departments [43]. In Table 1 are resumed the principal characteristics of several methods of Diuresis Renography.

In conclusion, the F+10(sp) method is safe, time-saving, well tolerated, easy to perform and gives information about baseline renal function (Table 2). This protocol avoids the side effects of other methods like diuresis-induced hypotension, bladder filling or disruption of the test because of voiding, without need of bladder catheterization. This method results in better patient compliance and a more accurate evaluation of urinary drainage and might be a valuable tool for urologists in the diagnosis of obstructive nephropathy and follow up of surgical treatments in urological cancer patients.

## Figures and Tables

**Table 1:** Timeline of Diuresis Renography: A comparison between several methods: F + 20 = furosemide injected 20 min after radiotracer injection; F-15 = furosemide injected 15 min prior radiotracer injection; F0 = furosemide injected contemporary of radiotracer injection; F + 2 = furosemide injected 2 min after radiotracer injection; F + 10(sp) = furosemide injected 10 min after radiotracer injection, in seated position (sp); PV = post-bladder voiding. (For interpretation of the references to colour in this table, the reader is referred to the web version of this article.)

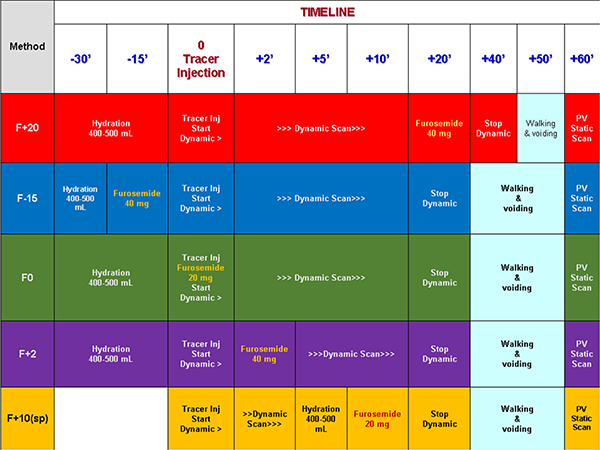

**Table 2:** Timeline of Diuresis Renography using the F + 10(sp) method. The diuresis renography demonstrated a dilation of the left (LT) kidney and an obstruction of right (RT) kidney. BKG = background IV = intravenous injection. (For interpretation of the references to colour in this table, the reader is referred to the web version of this article.)